# Microblading reaction as a manifestation of systemic sarcoidosis: two case reports and a review of the literature

**DOI:** 10.1186/s13256-024-04439-w

**Published:** 2024-04-24

**Authors:** Eva Klara Merzel Šabović, Mateja Starbek Zorko, Violeta Hosta, Borut Žgavec, Vid Bajuk

**Affiliations:** 1 Dermatology and Venereology Clinic, University Medical Centre Ljubljana, Gradiskova 10, 1000 Ljubljana, Slovenia; 2 Medical Faculty Ljubljana, University of Ljubljana, Ljubljana, Slovenia

**Keywords:** Sarcoidosis, Microblading, Dermal infiltration, Aesthetic procedure, Case report

## Abstract

**Background:**

Sarcoidosis is a multisystemic disease characterized by granulomatous inflammation. Sarcoidosis often poses a diagnostic challenge owing to its nonspecific or mild clinical features. In 20–35% of cases, sarcoidosis initially presents on skin. However, skin lesions commonly mimic dermatological conditions. Therefore, it is important to not underestimate the skin manifestations and perform histopathological examinations to make a timely diagnosis.

**Case presentation:**

We present two cases of 33-year-old Caucasian female patients with orange–red macules and plaques that developed in the eyebrow area 1 and 6 years after microblading, respectively. Histopathological examination confirmed a diagnosis of sarcoidosis. The lymph nodes and lungs were also affected in both patients.

**Conclusion:**

Our two reports suggest that an esthetic procedure involving dermal or subcutaneous injection of foreign materials can trigger the development of cutaneous and systemic sarcoidosis. However, this relationship has not been described yet. Physicians should, therefore, be aware of this complication to properly evaluate and treat such patients in a timely manner.

## Introduction

Sarcoidosis is a complex disease with unknown etiology and pathogenesis. Several factors may contribute to the development of sarcoidosis, such as a genetic predisposition with certain human leukocyte antigen (HLA) genotypes [1] and/or environmental factors, such as mycobacterial or propionibacterial organisms [2]. In addition, there is evidence suggesting an autoimmune or immune-mediated genesis of the disease [3]. In sarcoidosis, both the innate and adaptive immune systems are involved, highlighting the complexity of the pathogenesis of sarcoidosis [4]. Nevertheless, the specific causes that trigger the development of the granulomatous/fibrotic process in sarcoidosis remain unknown [4]. The prevalence of sarcoidosis varies by geographic location, with Scandinavian countries having the highest prevalence of 140–160 per 100,000 persons, while the prevalence in South Korea, Taiwan, and Japan is 1–5 per 100,000 [5].

Sarcoidosis is most commonly present in a mild form. However, in some cases, it can also be lethal, primarily because of the cardiac involvement. It is characterized by noncaseating granulomatous inflammation, which most commonly affects the lungs, lymph nodes, eyes, and skin [6]. The etiology of this condition is still not entirely understood. Sarcoidosis is believed to be triggered by exposure to extrinsic antigens in immunogenetically susceptible individuals [7]. According to previous studies, extrinsic antigens are the most common microbial agents and environmental substances [8]. In the developed world, there are growing reports of esthetic procedures with dermal infiltration, which might act as an extrinsic agent triggering sarcoidosis in predisposed individuals [9–16]. Here, we present two cases of a 33-year-old female patient who presented with orange–red maculopapular lesions in the eyebrow area after microblading. To date, only one case of cutaneous sarcoidosis after microblading has been reported [17]. However, no cases of systemic sarcoidosis after microblading, as in our two cases, have been reported.

## Case presentation

### Case 1

**Fig. 1:**
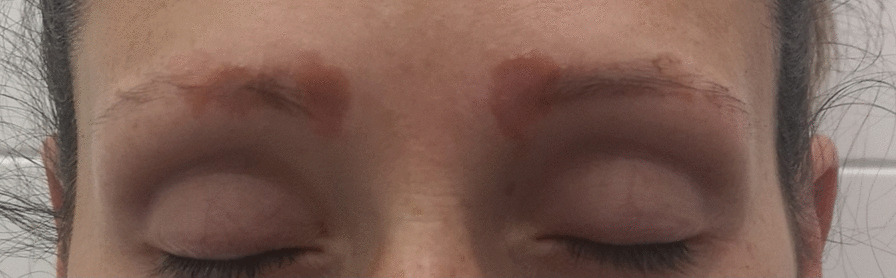
The first case of a female patient with mildly infiltrated erythematous plaques in the eyebrow area with a typical orange hue

**Fig. 2:**
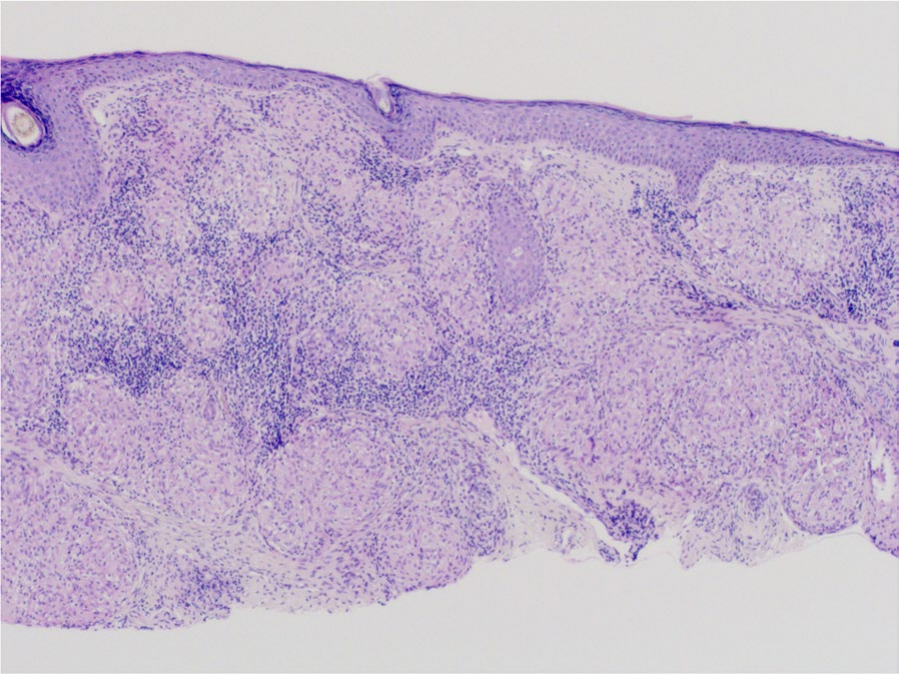
Histopathological examination of the first patient showing granulomatous dermatitis with epithelioid (sarcoid) granulomas

**Fig. 3:**
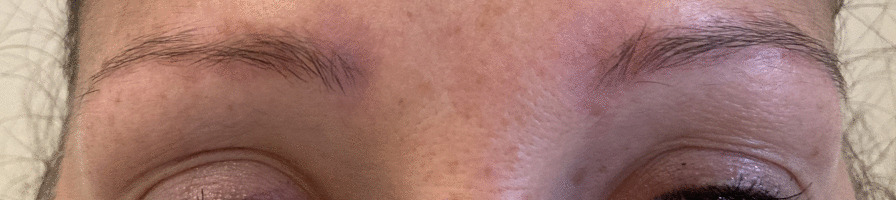
Resolution of cutaneous lesions in the first patient after 3 months of treatment with systemic corticosteroids

A 33-year-old Caucasian female patient presented with orange–red slightly infiltrated plaques in the eyebrow region that had been present for 3 years (Fig. 1). The plaques occurred 1 year after semipermanent tattooing—microblading, which had been performed only once. The skin was thoroughly examined, and the skin lesions were present only in the eyebrow area. First, allergic contact dermatitis was suspected and excluded using patch testing. Owing to the specific color of the lesions, a sarcoid granulomatous reaction was suspected. A thorough occupational and travel history was obtained, with no evidence of external influences that might cause sarcoidosis. Family history was negative for sarcoidosis. Her medical history was otherwise unremarkable. However, she reported a breast augmentation procedure that had been performed two years before the appearance of the skin lesions. A biopsy of the skin lesions revealed granulomatous dermatitis with epithelioid (sarcoid) granulomas (Fig. 2). Accordingly, sarcoidosis was suspected and the patient was referred for laboratory workup and chest radiography. Laboratory workup results were unremarkable with normal C-reactive protein (< 5 mg/L), normal angiotensin-converting enzyme (0.50 µkat/L), normal levels of serum calcium (2.37 mmol/L), magnesium (0.71 mmol/L), potassium (4.9 mmol/L), and sodium (137 mmol/L). However, chest radiography revealed hilar lymphadenopathy and reticulonodular opacities in the lungs, consistent with grade II sarcoidosis. She denied any pulmonary symptoms. Additionally, the patient was examined for other manifestations of sarcoidosis, which were excluded by other specialists. She was referred to a pulmonologist who performed an additional examination that revealed the progressive nature of pulmonary sarcoidosis with initial fibrosis. The patient required systemic treatment with prednisolone at an initial dose of 16 mg daily. After three months of systemic treatment with prednisolone 16 mg daily and topical treatment with momethasone 1 mg/g cream twice weekly, resolution of the cutaneous lesions was observed (Fig. 3). During the 2-year follow-up period in which she received continuous systemic prednisolone, we observed no recurrence of the skin lesions despite the gradual reduction of the prednisolone dose to 6 mg every other day. In addition, the reticular pulmonary changes regressed.

### Case 2

**Fig. 4:**
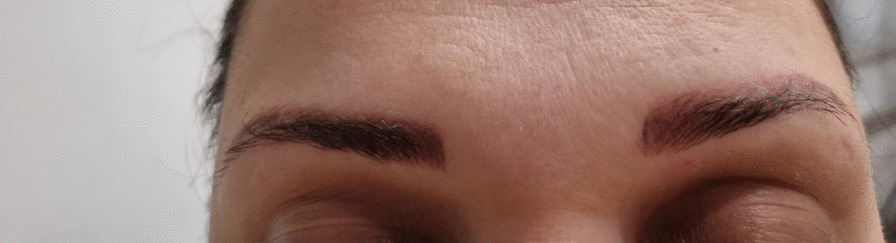
The second case of a female patient with mildly infiltrated erythematous plaques in the eyebrow area with a typical orange hue

**Fig. 5:**
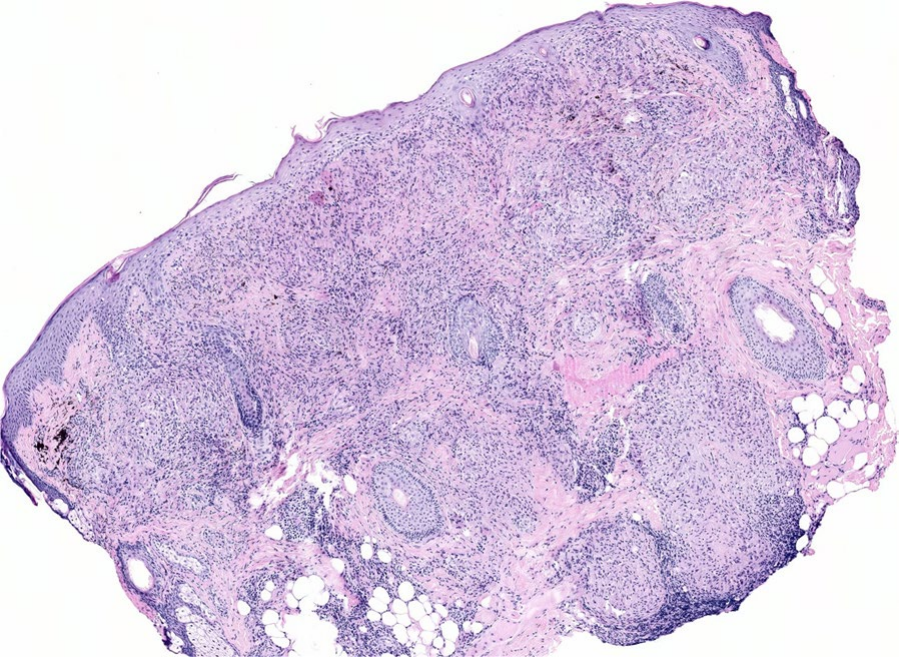
Histopathological examination of the second patient showing granulomatous dermatitis with epithelioid (sarcoid) granuloma

A 33-year-old Caucasian female patient presented with orange–red mildly infiltrated plaques in the eyebrow area that lasted for three months (Fig. 4). Upon thorough examination no other skin lesions were observed. She underwent microblading for 6 consecutive years, after which skin lesions developed. As in the first case, thorough occupational and travel histories were taken, and were unremarkable for external agents that could trigger sarcoidosis. Family history was also negative for sarcoidosis. Owing to the red–orange color of the lesions, a sarcoid granulomatous reaction was suspected and confirmed upon histopathological examination (Fig. 5). Laboratory workup revealed elevated erythrocyte sedimentation (34 mm/h), elevated C-reactive protein (13 mg/L), elevated angiotensin-converting enzyme (1.46 µkat/L), elevated chitotriosidase (480 nmol/h ml), and hypercalciuria (4.7 mmol/L) with a urine calcium/creatinine ratio of 0,96. Normal levels of serum calcium (2.23 mmol/L), magnesium (0.71 mmol/L), potassium (4.4 mmol/L), and sodium (139 mmol/L) were observed, however level of phosphate (0.50 mmol/L) was decreased. Furthermore, chest radiography revealed hilar lymphadenopathy and reticulonodular opacities in the lungs, which was consistent with sarcoidosis. The patient was referred to a pulmonologist who performed additional examinations and confirmed grade II sarcoidosis. However, owing to its non-progressive nature without fibrosis, treatment was not initiated at the time. Additional workup was performed for other manifestations of sarcoidosis; however, in addition to the skin, lungs, lymph nodes, and endocrine organs (hypercalciuria), other organs were not involved. Moreover, a high-resolution computed tomography scan performed 6 months after the initial diagnosis revealed resolution of sarcoidosis lesions in the lungs. For cutaneous involvement, she was prescribed mometasone furoate cream once daily for 10 days and then twice weekly as a maintenance treatment. However, no improvement was initially noted in the eyebrow area. After remission of pulmonary sarcoidosis and upon a 1-year follow-up visit, significant improvement of the skin was observed—only slight erythema of eyebrows persisted.

## Discussion

Sarcoidosis is a disease that remains largely unknown. The most frequent question bothering both physicians and patients is its trigger. This question is particularly important because sarcoidosis is not very rare [5]. Sarcoidosis is more common in women, and its onset is often in the third or fourth decade of life [7].

Diagnosis is often delayed owing to nonspecific or mild clinical features. Sarcoidosis affects multiple organs. The most frequently involved organs were the lungs (90% of patients), lymph nodes, and eyes (60%). Sarcoidosis can also affect other organs, such as the liver and spleen, heart (20–27%), endocrine and exocrine organs (hypercalciuria and hypercalcemia in 10% of patients), nervous system (10%), bones (1–13%), upper respiratory tract (2%), and kidneys owing to renal calculi formation [18]. Skin involvement is present in 20–35% of patients [7].

Dermatologists are in a unique position to make a timely diagnosis since skin manifestations are often a presenting feature. Sarcoidosis can present with specific or nonspecific skin lesions. Specific lesions show granulomatous inflammation on biopsy, whereas nonspecific lesions manifest only as inflammatory reactions without granuloma formation [6]. The most common skin manifestations include maculopapular, plaque-like, and subcutaneous lesions and erythema. It may also present as lupus pernio or with rarer, nonspecific forms, such as psoriasiform, annular, lichenoid, verrucous, photodistributed, and ichthyosiform [7].

Sarcoidosis is thought to develop in immunogenetically susceptible individuals after exposure to extrinsic antigens. Extrinsic antigens are the most common microbial or environmental triggers [7]. Mycobacterium and Propionibacterium are the most common microbial triggers [8]. Among the environmental triggers, people who worked in areas with mold or were exposed to insecticides were more likely to develop sarcoidosis [19]. Both patients denied having lived or worked in places with mold and had unremarkable travel histories. The first patient worked as a cashier, and the second as a nurse.

In modern times, people, especially in developed countries, are less likely to be exposed to potential contaminants at work or at home but are more likely to undergo esthetic procedures. According to increasing reports in literature, esthetic procedures could serve as an extrinsic trigger for the clinical manifestation of sarcoidosis in immunogenetically susceptible individuals, as suspected in our two cases. Granulomatous reactions have been observed after tattooing [10, 14–16], microneedling [11–13], injection of botulinum toxin [20, 21], and injection of dermal fillers [9, 22]. Interestingly, there is also a case report of cutaneous and pulmonary sarcoidosis after breast augmentation [23].

Microblading is a popular esthetic procedure used primarily for eyebrow restoration; however, it has also gained popularity for some dermatologic conditions, including alopecia totalis and madarosis owing to hypothyroidism or chemotherapy. Microblading is a superficial micropigmentation procedure in which pigments are introduced deep into the papillary dermis. The results are semipermanent and last 12–18 months [24]. Granulomas rarely develop at microblading sites [24]. In a recent review article, 21 cases of sarcoidosis were noted after permanent tattooing but only 1 after microblading. In the case of sarcoidosis that developed after microblading, only the skin was affected [17]. Interestingly, both patients were found to have systemic sarcoidosis. Therefore, microblading probably triggered the development of sarcoidosis in both patients. As the pigment deposit in microblading is shallower and presumably not dispersible compared to classic tattooing, resolution of granulomatous inflammation is probably more likely than in sarcoidosis after classical tattooing [17, 24].

Common to all esthetic procedures associated with sarcoidosis appears to be infiltration of the dermis or subcutis with foreign substances that may serve as triggers for sarcoidosis. However, sarcoidosis is not always the cause of granulomatous reactions. Therefore, we propose the following clinical recommendation: when a patient presents with chronic lesions at the site of injection of foreign material, especially with skin lesions of orange–red color, the patient should be appropriately evaluated for sarcoidosis. Such an examination should initially include a biopsy, which should then guide us further toward sarcoidosis or another diagnosis. If the histopathologic examination reveals a granulomatous inflammatory infiltrate, we should exclude other diseases consisting of granulomatous inflammation, such as foreign body granulomas, infectious granulomas, and interstitial granulomatous dermatitis [7, 25].

Treatment of patients with granulomatous reactions after dermal procedures consists of topical or oral corticosteroid therapy, or treatment with other oral immunosuppressants [14]. In patients with mild cutaneous sarcoidosis, topical or intralesional corticosteroids are used as the first-line therapy. In rapidly progressive extensive sarcoidosis or in cases of ineffective topical therapy, patients are treated with oral corticosteroids. Tumor necrosis factor-α (TNF-α) inhibitors are beneficial for treating recurrent skin lesions [26]. However, TNF-α inhibitors can sometimes even induce a paradoxical reaction with the development of a sarcoidosis-like reaction [27]. Other treatment options include antimalarials, methotrexate, tetracycline antibiotics, mycophenolate mofetil, and thalidomide [25]. Recently, treatment with Janus kinase inhibitors has been proposed to be effective [28, 29]. Case studies have also shown a beneficial effect of treating skin lesions with psoralen plus ultraviolet A light phototherapy, photodynamic therapy, pulsed dye lasers, and CO_2_ lasers [7].

Interestingly, our two cases differed significantly in the time interval between the triggering event (microblading procedure) and onset of cutaneous sarcoidosis. In the first patient, sarcoidosis manifested on the skin 1 year after the microblading procedure, whereas in the second patient, it occurred 6 years after the initial microblading procedure, which was followed by several repeated procedures in the following years. Our two cases raised some relevant questions and assumptions, even though they could not be fully explained and answered. For example, sarcoidosis may be triggered in susceptible individuals when a certain threshold for the accumulation of a foreign substance (ink) is reached. Implantation of other foreign substances, such as breast implants, as in our first patient, could increase susceptibility to cutaneous sarcoidosis triggered by microblading. This could explain why the first patient developed sarcoidosis after one procedure, whereas the second patient developed sarcoidosis after six microblading procedures. In addition, a progressive course of sarcoidosis with pulmonary fibrosis in the first patient could be a coincidence; however, it could be owing to the additive deleterious effects of multiple implanted foreign substances in addition to the effect of the long-term presence of the triggering substance, that is, the deposited skin pigment, in subsequent years. It is also unknown whether time-dependent pigment dispersion plays an important role. Therefore, long-term follow-up is required. It is difficult to predict whether skin changes will recur after discontinuation of systemic corticosteroid therapy in the first patient because local corticosteroids initially did not eliminate cutaneous sarcoidosis in the second patient. In this case, spontaneous remission of sarcoidosis was most likely the reason for the resolution of skin symptoms, which according to some reports, may be due to the high release of transforming growth factor β from alveolar macrophages [30]. However, this observation suggests that systemic corticosteroid treatment is required to treat cutaneous sarcoidosis. Therefore, more real-world data are required. However, the possibility of preexisting sarcoidosis and microblading as triggers of skin sarcoidosis in our two cases cannot be neglected. However, this assumption does not diminish the role of microblading in sarcoidosis development or progression.

In addition, it will be interesting to continue monitoring both patients, especially the first one treated with systemic corticosteroids because of progressive pulmonary involvement, to determine whether cutaneous lesions recur after treatment cessation. Depending on the transient effect of the microblading procedure, one might predict that the skin lesions will not recur; however, the pigment remains in the body because it is not eliminated from the tissues. It is difficult to predict whether the dispersed pigment acts as a trigger for sarcoidosis or not.

Because sarcoidosis most commonly affects women between the ages of 30 and 40 years, it is important to emphasize that physicians should educate all patients with sarcoidosis about potential skin complications after esthetic procedures involving infiltration of the dermis or subcutis. Other options, such as superficial peels or laser treatment, should be considered. However, further studies are needed to provide practical recommendations for esthetic procedures in patients with sarcoidosis. In addition, physicians and estheticians who perform esthetic procedures should be aware of the risk of sarcoidosis in patients with a predisposition. Prior to such procedures, patients should be adequately informed of this risk despite its rarity.

## Conclusion

As our two cases also demonstrated, dermal or subcutaneous infiltration with foreign material during esthetic procedures can trigger the manifestation of sarcoidosis. When esthetic procedures become increasingly popular, patients with chronic lesions at the injection site of the foreign material, especially macular, papular, or nodular lesions of orange–red color, should be appropriately evaluated for possible sarcoidosis. Such an examination should initially include a biopsy, which should guide the physician in the direction of either sarcoidosis or another diagnosis. In addition, all patients with sarcoidosis should be educated and warned of such complications before undergoing esthetic procedures.

## Data Availability

Available.
